# DNA Methylation Mediates lncRNA2919 Regulation of Hair Follicle Regeneration

**DOI:** 10.3390/ijms23169481

**Published:** 2022-08-22

**Authors:** Bohao Zhao, Jiali Li, Ming Liu, Naisu Yang, Zhiyuan Bao, Xiyu Zhang, Yingying Dai, Jiawei Cai, Yang Chen, Xinsheng Wu

**Affiliations:** 1 College of Animal Science and Technology, Yangzhou University, Yangzhou 225009, China; 2 Joint International Research Laboratory of Agriculture & Agri-Product Safety, Yangzhou University, Yangzhou 225009, China

**Keywords:** hair follicle cycle, lncRNA, lncRNA2919, rabbit model, DNA methylation, EGR1

## Abstract

Hair follicles (HFs) are organs that periodically regenerate during the growth and development of mammals. Long non-coding RNAs (lncRNAs) are non-coding RNAs with crucial roles in many biological processes. Our previous study identified that lncRNA2919 is highly expressed in catagen during the HF cycle. In this study, the in vivo rabbit model was established using intradermal injection of adenovirus-mediated lncRNA2919. The results showed that lncRNA2919 decreased HF depth and density and contributed to HF regrowth, thereby indicating that lncRNA2919 plays a negative role in HF regeneration. Moreover, methylation levels of the lncRNA2919 promoter at different HF cycle stages were detected through bisulfite sequencing. The key CpG site that negatively correlates with lncRNA2919 expression during the HF cycle was identified. 5-Aza-dc-induced demethylation upregulated lncRNA2919 expression, and the core promoter region of lncRNA2919 was verified on the basis of luciferase activity. Furthermore, we found that DNA methylation could prevent the binding of EGR1 to the lncRNA2919 promoter region, thereby affecting the transcriptional expression of lncRNA2919. Collectively, DNA methylation inhibits the transcriptional expression of lncRNA2919, which plays a vital role in the HF cycle and HF regrowth. These findings contribute to the basic theory of epigenetics in HF biology and provide references for further research in HF disease treatment and animal wool production.

## 1. Introduction

Hair follicles (HFs) are unique, complex organs that periodically regenerate during growth and development processes in mammals, and they undergo continuous cycling, including growth (anagen), regression (catagen), and quiescence (telogen) [1,2]. Because of their high self-renewal ability and obvious structural characteristics, HFs could act as a good scientific model for explaining various aspects of cellular physiology, such as cell proliferation, differentiation, and apoptosis [3,4]. Long non-coding RNAs (lncRNAs) are non-coding RNAs that are more than 200 bp long and generally do not have the ability to encode proteins. lncRNAs play critical roles in many biological processes by regulating cellular activities, gene expression, protein complex formation, and chromatin states [5,6,7]. In HF biology, many lncRNAs participate in the regulation of HF growth and development. For example, PlncRNA-1 promoted the proliferation and differentiation of HF stem cells (HFSCs) by regulating the TGF-β1-mediated Wnt/β-catenin signaling pathway [8]. lncRNA-PCAT1 maintained the proliferation and activity of dermal papilla cells (DPCs) and promoted HF regeneration by regulating the PCAT1/miR-329/Wnt10b axis [9]. In our previous study, lncRNAs related to the HF growth and cycle were screened and lncRNA2919, a key lncRNA, was revealed to be significantly expressed during the HF cycle [10]. Therefore, whether lncRNA2919 regulates HF regeneration was investigated in an animal model of HF synchronization by subcutaneously injecting adenovirus-mediated lncRNA2919 in Angora rabbits.

DNA methylation could enrich the CpG island of the promoter and enhancer and may affect the binding sites of transcription factors (TFs) and regulate the transcriptional activation of transcripts [11]. Transcriptional expression of lncRNA is regulated by DNA methylation. A significantly high methylation level could inhibit the expression of lncRNA MEG3, which plays a vital role in esophageal squamous carcinoma [12]. An abnormal level of methylation in the lncRNA uc.167 promoter region suppressed myocardial cell differentiation [13]. Abnormal DNA demethylation could activate lncRNA SNHG12 expression through the binding of the TF SP1 in glioblastoma multiforme [14]. Moreover, different DNA methylation levels and lncRNA expression levels have been determined during different HF morphogenesis stages [15]. DNA methylation in the promoter of lncRNA H19 could regulate its transcriptional expression during different stages of the HF cycle [16]. During anagen to telogen, promoter methylation led to changes in the transcriptional expression of lncRNA HOTAIR in the secondary HF of cashmere goats [17]. Thus, whether epigenetic regulation of DNA methylation in the lncRNA2919 promoter could influence HF regeneration was investigated.

In the current study, the role of lncRNA2919 in regulating HF periodical regeneration was revealed using an animal model of HF synchronization. Subsequently, DNA methylation levels in the lncRNA2919 promoter during the HF cycle were investigated; demethylation and TF regulated the transcriptional expression of lncRNA2919. These findings contribute to filling the gaps due to insufficient study of epigenetics in HF biology and provide theoretical references for further research in animal wool production and human hair disease therapy.

## 2. Results

### 2.1. LncRNA2919 Regulates HF Regeneration in the In Vivo Rabbit Model

**Figure 1:**
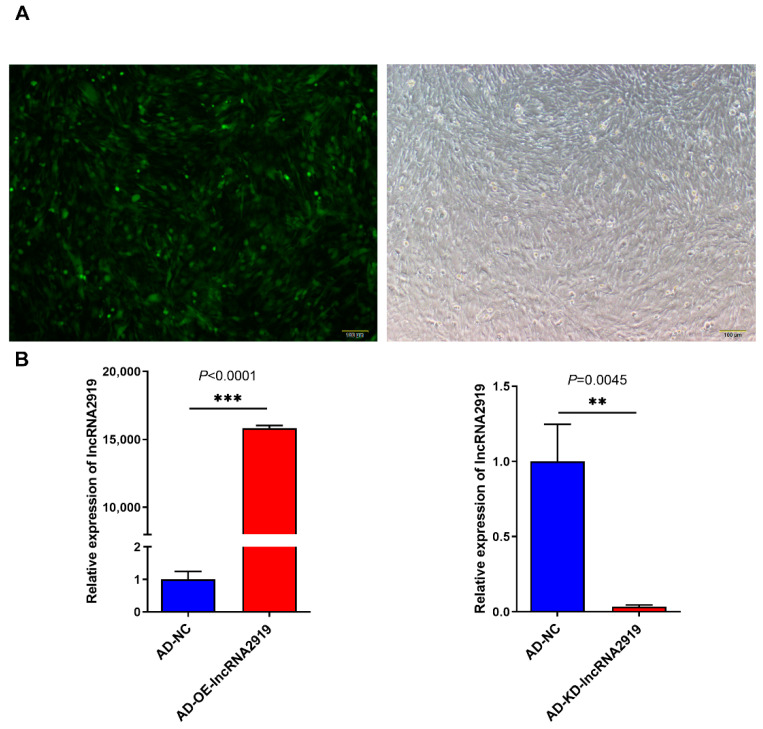
Infection of AD-lncRNA2919 in DPCs. (**A**) Efficiency of infection of AD-lncRNA2919 in DPCs. Scar bar = 100 μm. (**B**) The lncRNA2919 expression level in the AD-OE-lncRNA2919 and AD-KD-lncRNA2919 groups. Data are presented as mean ± SEM. The two-tailed paired *t*-test was used for data analyses. For significance, ** *p* < 0.01, *** *p* < 0.001.

To explore the effect of lncRNA2919 on HF cyclic regeneration in rabbits, the adenovirus infection system was constructed to upregulate or downregulate lncRNA2919 expression. The qRT-PCR result showed that lncRNA2919 was significantly upregulated in the DPCs compared with the rabbit skin fibroblasts (RAB-9) (Figure S1). Fluorescence microscopy showed that adenovirus infection in DPCs was successful (Figure 1A). qRT-PCR showed that adenovirus-overexpression (AD-OE)-lncRNA2919 infection in DPCs could significantly upregulate lncRNA2919 expression, and adenovirus-knockdown (AD-KD)-lncRNA2919 infection could downregulate lncRNA2919 expression (Figure 1B).

**Figure 2:**
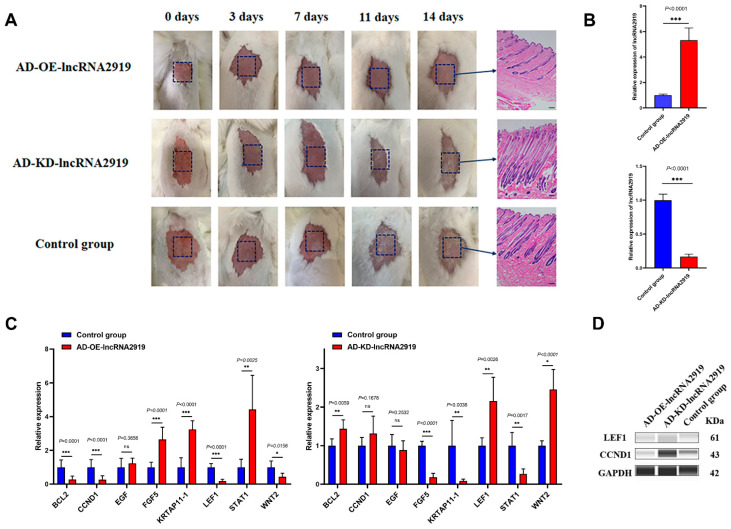
AD-lncRNA2919 regulates HF regeneration in Angora rabbits. (**A**) Morphological observation of rabbit skins after subcutaneous injection in the AD-OE-lncRNA2919, AD-KD-lncRNA2919, and control groups. Scar bar = 200 μm. (**B**) lncRNA2919 expression in AD-OE-lncRNA2919 and AD-KD-lncRNA2919 after the subcutaneous injection of adenovirus in Angora rabbits. (**C**) lncRNA2919 regulated the HF cycle- and growth-related genes in Angora rabbits. (**D**) lncRNA2919 regulated the HF cycle- and growth-related proteins in Angora rabbits. Data are presented as mean ± SEM. The two-tailed paired *t*-test was used for data analyses. For significance, * *p* < 0.05, ** *p* < 0.01, *** *p* < 0.001, ns indicates not significant.

In in vivo experiments, AD-OE-lncRNA2919 and AD-KD-lncRNA2919 were subcutaneously injected into the dorsal skin of the HF synchronization model of Angora rabbits. The results showed that lncRNA2919 could reduce the density and depth of HF, delay hair shaft growth, extend the transition from telogen to anagen, and inhibit HF regeneration (Figure 2A). The qRT-PCR results showed that AD-OE-lncRNA2919 significantly upregulated lncRNA2919 expression, but AD-KD-lncRNA2919 significantly downregulated the expression in rabbit dorsal skins (Figure 2B). lncRNA2919 significantly upregulated *FGF5*, *KRTAP11-1*, and *STAT1* mRNA expression, whereas it significantly downregulated *BCL2*, *CCND1*, *LEF1*, and *WNT2* mRNA expression (Figure 2C). lncRNA2919 regulated LEF1 and CCND1 protein expression (Figure 2D), which indicated that lncRNA2919 plays a negative role in HF growth and development, and HF regeneration.

### 2.2. DNA Methylation Level of LncRNA2919 during the HF Cycle

**Figure 3:**
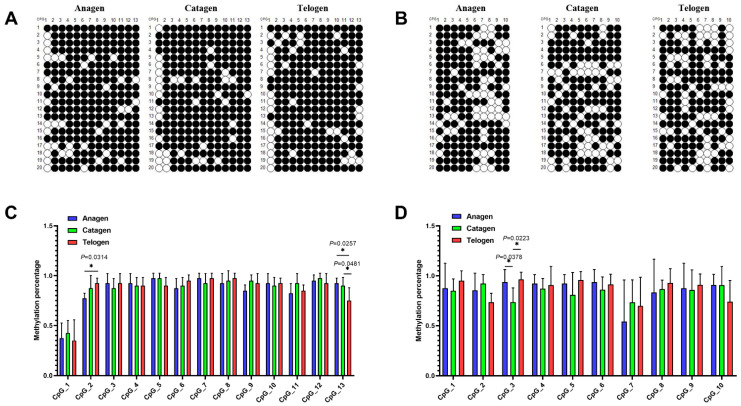
DNA methylation regulates the transcriptional expression of lncRNA2919. (**A**) DNA methylation detection of CpG island 1 in the lncRNA2919 promoter region during the HF cycle in Angora rabbits. (**B**) DNA methylation detection of CpG island 2 in the lncRNA2919 promoter region during the HF cycle in Angora rabbits. (**C**) Methylation percentage of CpG island 1 in the lncRNA2919 promoter region during the HF cycle. (**D**) Methylation percentage of CpG island 2 in the lncRNA2919 promoter region during the HF cycle. Data are presented as mean ± SEM. One-way ANOVA with adjusted multiple comparisons was used for data analyses. For significance, * *p* < 0.05.

To explore the upstream regulatory mechanism of lncRNA2919, the DNA methylation region of the lncRNA2919 promoter was predicted, and it contained two CpG islands (CpG island 1, −1075 to −781; CpG island 2, −1201 to −1101). The DNA methylation level of lncRNA2919 was detected during the HF cycle (anagen, catagen, and telogen) through bisulfite sequencing (BS) (Figure 3A,B). In CpG island 1, most CpG sites were hypermethylation sites, except for the CpG_1 site. The methylation level of the CpG_2 site was significantly higher in telogen than in anagen, while that of the CpG_13 site was significantly higher in anagen and catagen than in telogen (Table S1, Figure 3C). In CpG island 2, most CpG sites were hypermethylation sites, except for the CpG_7 site. The methylation level of the CpG_3 site was significantly higher in catagen than in anagen (Table S2, Figure 3D). In addition, the methylation levels of CpG sites in the lncRNA2919 promoter and the lncRNA2919 expression level during the HF cycle were analyzed through correlation analysis. We found that the CpG_3 site in CpG island 2 was negatively correlated with lncRNA2919 expression in different HF cycle stages (Pearson correlation coefficient was −0.99).

### 2.3. DNA Methylation Regulates LncRNA2919 Transcriptional Expression

**Figure 4:**
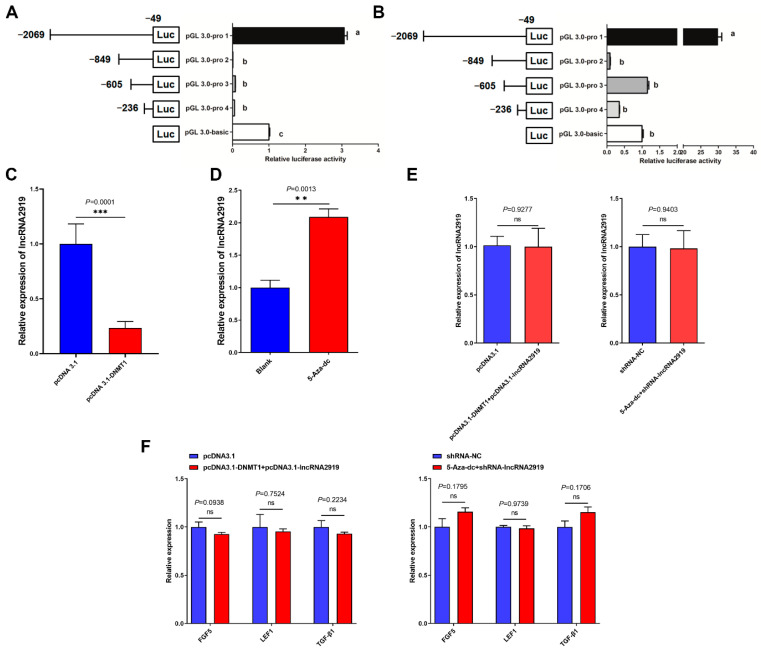
Methylation regulates lncRNA2919 transcriptional expression. (**A**) The detection of luciferase activity in the lncRNA2919 promoter region. (**B**) The detection of luciferase activity in the lncRNA2919 promoter region by demethylation. (**C**) The lncRNA2919 expression level was regulated by pcDNA3.1-DNMT1. (**D**) The lncRNA2919 expression level was regulated by 5-Aza-dc. (**E**) The detection of lncRNA2919 expression whether pcDNA3.1-DNMT1 and 5-Aza-dc could rescue the effect of overexpression and knockdown of lncRNA2919 in DPCs. (**F**) The detection of HF cycle-related gene expression whether pcDNA3.1-DNMT1 and 5-Aza-dc could rescue the effect of overexpression and knockdown of lncRNA2919 in DPCs. Data are presented as mean ± SEM. The two-tailed paired *t*-test was used for data analyses. One-way ANOVA with adjusted multiple comparisons was also used for data analyses. Different letters indicate significant differences (*p* < 0.05). For significance, ** *p* < 0.01, *** *p* < 0.001, ns indicates not significant.

To further explore whether methylation regulates lncRNA2919 expression, DPCs were demethylated using the methyltransferase inhibitor 5-Aza-dc, which could significantly inhibit *DNMT1* mRNA expression (Figure S2A). The transfection of pcDNA3.1-DNMT1 could significantly upregulate DNMT1 mRNA expression (Figure S2B). Then, the relationship between DNA methylation and promoter activities was investigated. Luciferase reporter assays showed that −2069 to −849 loci in the lncRNA2919 promoter was the region with the highest luciferase activity (Figure 4A). We detected luciferase activity in −2069 to −849 loci after demethylation using 5-Aza-dc. The luciferase activity in the core promoter region was significantly upregulated (Figure 4B), and the CpG_3 site in CpG island 2 was located in this region. Additionally, we detected lncRNA2919 expression after treatment of pcDNA3.1-DNMT1 and 5-Aza-dc through qRT-PCR. The results showed that pcDNA3.1-DNMT1 could significantly downregulate lncRNA2919 expression (Figure 4C). 5-Aza-dc significantly increased lncRNA2919 expression in DPCs (Figure 4D). Furthermore, DNMT1 overexpression was found to rescue lncRNA2919 expression after lncRNA2919 overexpression. Demethylation rescued lncRNA2919 expression after lncRNA2919 knockdown in DPCs (Figure 4E). DNMT1 overexpression could rescue the mRNA expression level of *FGF5*, *LEF1,* and *TGF-β1* after lncRNA2919 expression. *FGF5*, *LEF1,* and *TGF-β1* mRNA expression showed no significant difference between the co-transfection group of 5-Aza-dc and shRNA-lncRNA2919 and the control group in DPCs (Figure 4F).

### 2.4. TF EGR1 Regulates LncRNA2919 Transcriptional Expression

**Figure 5:**
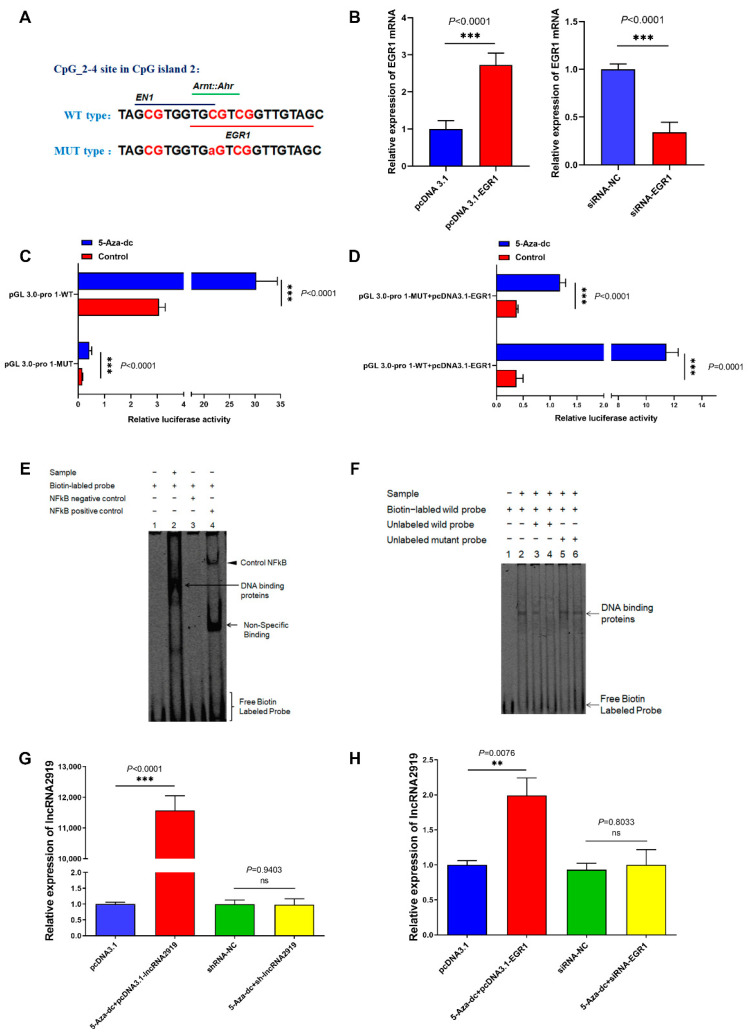
TF EGR1 regulates the transcriptional expression of lncRNA2919. (**A**) The TF prediction of CpG_2 to CpG_4 in CpG island 2 of the lncRNA2919 promoter region. (**B**) The EGR1 mRNA expression level after EGR1 overexpression and knockdown in DPCs. (**C**) Luciferase activities were detected after the transfection of the wild-type and mutant-type vectors of EGR1 after demethylation. (**D**) Luciferase activities were detected after co-transfections of the wild-type vector and pcDNA3.1-EGR1, and the mutant-type vector and pcDNA3.1-EGR1 after demethylation. (**E**) The binding relationship between EGR1 and the lncRNA2919 promoter was verified through EMSA. (**F**) The binding relationship between EGR1 and the lncRNA2919 promoter was verified through competitive EMSA. The volume of unlabeled oligonucleotides was 33-fold higher than that of labeled oligonucleotides in the third and fifth lanes, and the volume of unlabeled oligonucleotides was 100-fold higher than that of labeled oligonucleotides in the fourth and sixth lanes. (**G**) The lncRNA2919 expression level was regulated by demethylation. (**H**) The lncRNA2919 expression level was regulated by demethylation and EGR1. Data are presented as mean ± SEM. The two-tailed paired *t*-test was used for data analyses. For significance, ** *p* < 0.01, *** *p* < 0.001, ns indicates not significant.

By the TF prediction of the CpG_3 site in CpG island 2, we identified that TF early growth response 1 (EGR1) was located in the region (Figure 5A). The overexpression and knockdown of *EGR1* mRNA were determined through qRT-PCR (Figure 5B). The results showed that EGR1 could specifically bind to the lncRNA2919 promoter region (−1121 to −1108) and significantly promote the activities of this region through demethylation, site mutation, and luciferase reporter assays (Figure 5C). EGR1 overexpression and demethylation could significantly upregulate lncRNA2919 transcriptional activities (Figure 5D). The sites in the lncRNA2919 promoter region for binding to EGR1 were further confirmed through the electrophoretic mobility shift assay (EMSA), which confirmed that EGR1 may bind to the lncRNA2919 promoter region (Figure 5E). Unlabeled EGR1 probes competitively disrupted the binding capacity, indicating that EGR1 may specifically bind to the lncRNA2919 promoter (Figure 5F). The rescue assays demonstrated that demethylation and pcDNA3.1-lncRNA2919 could significantly upregulate the lncRNA2919 expression level in DPCs (Figure 5G). DNA demethylation and TF EGR1 could significantly upregulate lncRNA2919 expression in DPCs (Figure 5H). These data revealed that DNA methylation regulated the binding relationship between TF EGR1 and the lncRNA2919 promoter region, regulating lncRNA2919 transcription and participating in the HF regeneration process.

## 3. Discussion

HF growth and development are mediated by the interaction of multiple biomolecules and regulation of the signaling pathway, which may allow a deep understanding of metabolism, endocrine regulation, angiogenesis, and neurogenesis [1,2,18,19,20,21]. As a class of non-coding RNA molecules, lncRNAs play vital roles in HF growth and development. For example, lncRNA XLOC_008679 could regulate the HF cycle by KRT35 in anagen [22], lncRNA H19 maintained the HF-inducing ability through the Wnt/β-catenin signaling pathway [23], and lncRNA5322 promoted HFSC proliferation and differentiation through the PI3K-AKT signaling pathway [24]. Our previous study identified that lncRNA2919 was significantly expressed in catagen during the HF cycle [10]. The prediction results showed that lncRNA2919 may regulate KRTAP11-1 through the co-expression/trans-regulatory relationship. KRTAP11-1 plays a crucial role in the assembly of keratin bundles, formation of hair fibers, and hair quality [25,26], thus indicating that lncRNA2919 may participate in the regulation of HF growth and development.

In previous studies, adenovirus-Shh injected intradermally in the skin of mice promoted hair regrowth [27], and the in vivo mouse model, established through intradermal injection of adenovirus-mediated Wnt10b, revealed the role of Wnt10b in HF regeneration [28]. In this study, AD-lncRNA2919 may have regulated HF regeneration in Angora rabbits when injected intradermally. lncRNA2919 blocked hair shaft regrowth and the initiation of new anagen, extending the transition from telogen to anagen, which suggests that lncRNA2919 plays a negative role in HF regeneration. Furthermore, lncRNA2919 could dysregulate HF cycle- and growth-related genes. It upregulated the expression of *FGF5* [29,30], *KRTAP11-1* [31], and *STAT1* [32], whereas it downregulated the expression of *LEF1* [33], *WNT2* [34], *BCL2* [35,36], and *CCND1* [37].

Moreover, lncRNA may act in a regulatory manner by modifying DNA methylation [38]. The high expression of the lncRNA 1810019D21Rik was negatively correlated with the DNA methylation level in breast tumors [39]. 5-Aza-dc could decrease the DNA methylation level in the promoter region of the lncRNA MEG3, thereby inhibiting cell proliferation and invasion of esophageal tumor cells [12]. DNA methylation inhibited lncRNA H19 and lncRNA-000133 expression, which were highly expressed in anagen during the HF cycle [24,40]. However, only few studies have deeply investigated the inherent regulatory mechanism between methylation and lncRNA during the HF cycle. In this study, the DNA methylation rate in the promoter region of lncRNA2919 in the different stages of the HF cycle was determined, and most CpG sites were hypermethylated in the two CpG islands during the HF cycle. With the correlation analysis between methylation levels of CpG sites in the lncRNA2919 promoter and the lncRNA2919 expression level during the HF cycle, we found that the key CpG site (CpG_3 site in CpG island 2) was negatively correlated with lncRNA2919 expression in different HF cycle stages. With the prediction of TFs located on the key CpG site, TF EGR1 was identified to specifically bind to the lncRNA2919 promoter region. EGR1 could regulate the expression of TGF-β1, PTEN, and p53, which play critical roles in biological processes [41,42,43], and EGR1 may play a positive role in the HF growth and cycle [44]. We found that EGR1 overexpression promoted the transcriptional expression of lncRNA2919 after demethylation, indicating that methylation restrained the binding of TF EGR1 in the lncRNA2919 promoter.

## 4. Materials and Methods

### 4.1. Animals

All animal experimental procedures in the study were approved by the Animal Care and Use Committee of Yangzhou University. Six-month-old Angora male rabbits were raised in the same living condition. The rabbits were anesthetized with Zoteil 50, and 1-cm^2^ dorsal skin samples were collected and stored at –70 °C for RNA and protein extraction for the follow-up experiment. A portion of the skin samples was fixed in 4% formaldehyde, and paraffin sections were stained with hematoxylin–eosin for histological observations. Iodine solution was applied to the wound to prevent bacterial infection.

### 4.2. Cell Culture

Rabbit skin fibroblasts (RAB-9 cells, ATCC CRL-1414) maintained in minimum essential medium (Gibco, Grand Island, NY, USA) containing 10% fetal bovine serum (Gibco, Grand Island, NY, USA) were used for the dual-luciferase assay. DPCs procured from our research group, which were separated from the rabbit HFs according to a previous study [45] and maintained in mesenchymal stem cell medium (MSCM, Sciencell, San Diego, CA, USA), were used for the biological function verification of lncRNA2919. For the cell transfection, Lipofectamine 2000 or 3000 (Invitrogen, Carlsbad, CA, USA) was used following the manufacturer’s instructions.

### 4.3. Plasmid Preparation, Small Interfering RNA, Short Hairpin RNA, Adenovirus Plasmids, Transfection

Adenovirus packaging of lncRNA2919 was performed by GenePharma Co., Ltd. (Shanghai, China). AD-OE-lncRNA2919 was the full-length sequence of lncRNA2919 constructed using the ADV-M11 vector, and the titer of AD-OE-lncRNA2919 was 1 × 10^12^ PFU/mL. AD-KD-lncRNA2919 (5′-GGATCAGGAAGAATGCCTTGC-3′) was constructed using the ADV-M3 vector. The titer of AD-KD-lncRNA2919 was 1 × 10^12^ PFU/mL. For the adenovirus experiment in DPCs, cells were grown in 24-well plates and the optimum dilution condition was identified. Adenovirus solution (10 μL) and polybrene (1 μL) were added to the cell suspension, and the cells were collected after 48 h for the further experiment. Total RNA was isolated from the rabbit skin using the RNAsimple total RNA Kit (Tiangen, Beijing, China) for constructing the overexpression vector. The PrimeScript 1st Strand cDNA Synthesis Kit (Takara, Dalian, China) was used for the synthesis of high-quality rabbit skin cDNA, and then PCR was performed using Phanta Max Super-Fidelity DNA Polymerase (Vazyme, Nanjing, China). Following the steps, the overexpression vectors of pcDNA3.1-EGR1 and pcDNA3.1-DNMT1 were constructed, and pcDNA3.1-lncRNA2919 was constructed using the full-length sequence of lncRNA2919 [46]. The short hairpin RNA (shRNA)-lncRNA2919 and small interfering RNA (siRNA)-EGR1 were designed and purchased from GenePharma Co., Ltd. (Shanghai, China). The primers for aforementioned procedures are listed in Table S3.

### 4.4. Subcutaneous Injection of Adenovirus in Angora Rabbit

For establishing the HF synchronization model, a part of Angora rabbit dorsal skin was shaved with electronic clippers when HF had entered into the new anagen stage as evident through the appearance of light pink skin and wool regrowth. Twelve rabbits were divided into three groups: OE group (50 μL AD-OE-lncRNA2919 + 150 μL physiological saline solution), KD group (50 μL AD-KD-lncRNA2919 + 150 μL physiological saline solution), and control group (200 μL physiological saline solution). The solutions were subcutaneously injected into the dorsal skin on days 0 and 7. Then, hair shaft regrowth, HF growth and development, and onset of the HF cycle were observed, and the skin samples were collected on the 14th day for further analysis.

### 4.5. RNA Isolation and Quantitative RT-PCR Analysis

According to the manufacturer’s instructions, the total RNA of cells and skins was isolated using the RNAsimple total RNA Kit (Tiangen, Beijing, China). For the verification of the mRNA expression level, the AceQ qPCR SYBR Green Master Mix (Vazyme, Nanjing, China) was used for quantitative mRNA level analysis. For the verification of the lncRNA expression level, cDNA synthesis was performed using the lnRcute lncRNA First-Strand cDNA Kit (Tiangen, Beijing, China), and then qRT-PCR was performed using the lnRcute lncRNA qPCR Kit (SYBR Green, Tiangen, Beijing, China). The qRT-PCR data were analyzed using QuantStudio 5 (Applied Biosystems), according to the manufacturer’s instructions. Relative expression levels of mRNA and proteins were calculated using the 2^−ΔΔCt^ method [47], and glyceraldehyde 3-phosphate dehydrogenase (GAPDH) was used as the reference gene. The specific primers are listed in Table S4.

### 4.6. Protein Simple Wes Western Blotting System

The rabbit skins were collected for estimating the protein expression level. RIPA lysis buffer (PPLYGEN, Beijing, China) was used for collecting protein lysates, and the protein concentration was measured using the Enhanced BCA Protein Kit (Beyotime, Shanghai, China). According to the manufacturer’s instructions, 0.5 μg/μL of the protein sample was used for detecting the protein expression level by using the automated Western blotting system (Protein Simple Wes) [48]. The figures of Wes analysis were showed in Supplementary file S1. The following antibodies were used: 1:100 anti-CCND1 mouse monoclonal antibody, anti-LEF1 rabbit polyclonal antibody (Proteintech, Wuhan, China), and 1:100 anti-GAPDH mouse monoclonal antibody (Abcam, Cambridge, MA, USA).

### 4.7. DNA Extraction and Bisulfite Sequencing PCR

DNA from the skin samples of Angora rabbits, including that from anagen, catagen, and telogen stages of the HF cycle, was isolated using the TIANamp Genomic DNA Kit (Tiangen, Beijing, China), bisulfate-modified, and subjected to BS analysis with EpiTaq HS (for bisulfite-treated DNA, Takara, Dalian, China) according to the manufacturer’s instruction. The primers were designed using Methyl Primer Express v1.0 (Table S5), and BS PCR data were analyzed using BiQ Analyzer software.

### 4.8. Dual-Luciferase Assay Verification of the Promoter Region

Segments of lncRNA2919 promoter sequences were cloned into pGL3-Basic vectors. TFs in the promoter region were predicted referring to the JASPAR database (http://jaspar.genereg.net/ (accessed on 1 November 2020)) [49]. The mutation type of TF binding sites was determined using Mut Express II Fast Mutagenesis Kit V2 (Vazyme, Nanjing, China), and the primers used are listed in Table S6. After cell transfection, luciferase activity was determined using the dual-luciferase reporter system (Promega, Madison, WI, USA), and firefly luciferase activity was normalized to the corresponding Renilla luciferase activity.

### 4.9. 5-Aza-Deoxycytidine-Induced Demethylation

The DNA methyltransferase inhibitor, 5-Aza-deoxycytidine (5-Aza-dc), was purchased from MedChemExpress (Monmouth Junction, NJ, USA) and dissolved in dimethyl sulfoxide (Sigma, St. Louis, MO, USA) at a concentration of 0.15 mM. The cells were cultured in 24-well plates with 0.15 mg/mL 5-Aza-dc for 24 h for the subsequent steps.

### 4.10. Electrophoretic Mobility Shift Assay

The nuclear extracts of DPCs were collected using Nuclear and Cytoplasmic Extraction Reagents (Viagene Biotech, Changzhou China), and EMSA was conducted using a biotin-labeled EMSA kit (Viagene Biotech, Changzhou China). The bands were visualized using CoolImger III (Viagene Biotech, Changzhou, China). The sequences of the wild-type and mutant probes are listed in Table S7.

### 4.11. Statistical Analysis

The mRNA or lncRNA relative expression level, methylation rates, and luciferase activity were analyzed using one-way ANOVA or two-tailed Student’s *t*-test with SPSS 25.0 (SPSS Inc., Chicago, IL, USA). For each analysis, a minimum of three biological replicates was used. All error bars in the results represent the mean ± SEM.

## 5. Conclusions

In conclusion, intradermal injection of AD-OE-lncRNA2919 could block hair shaft regrowth in the in vivo rabbit model, and thus, lncRNA2919 plays a negative role in HF regeneration. DNA methylation can prevent the binding of EGR1 to the lncRNA2919 promoter region, which influences the transcriptional regulation of lncRNA2919. Therefore, the methylation and lncRNA2919 regulation network is involved in the regulation of the HF cycle. The research provides a theoretical reference of how lncRNAs regulate HF regrowth and how DNA methylation alters lncRNA expression during the HF cycle, thereby filling the research gap in hair disease therapy and animal wool production.

## Data Availability

All data supporting our findings are included in the manuscript.

## Supplementary Materials

The following supporting information can be downloaded at: https://www.mdpi.com/article/10.3390/ijms23169481/s1.
