# Supplementary material for: DNA Methylation Mediates lncRNA2919 Regulation of Hair Follicle Regeneration
Source: Int J Mol Sci. 2022 Aug 22;23(16):9481. doi: 10.3390/ijms23169481 (PMC9408817; doi:10.3390/ijms23169481)
Supplement: Supplementary file 1 [file ijms-23-09481-s001.zip › Figure S1.pdf]

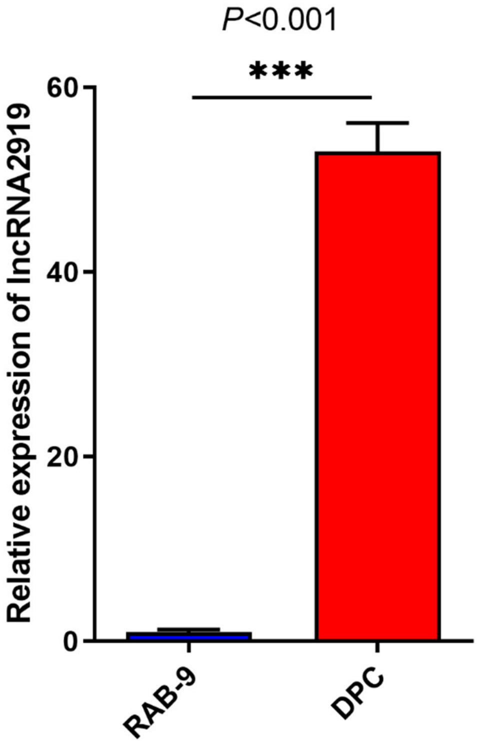

Figure S1. lncRNA2919 expression between rabbit DPC and RAB-9. Data are presented as mean ±SEM. A two-tailed paired t-test was used for data analyses. For significance, *** $P < 0.001$.
