# Supplementary figures and images for: DNA Methylation Mediates lncRNA2919 Regulation of Hair Follicle Regeneration
Source: Int J Mol Sci. 2022 Aug 22;23(16):9481. doi: 10.3390/ijms23169481 (PMC9408817; doi:10.3390/ijms23169481)

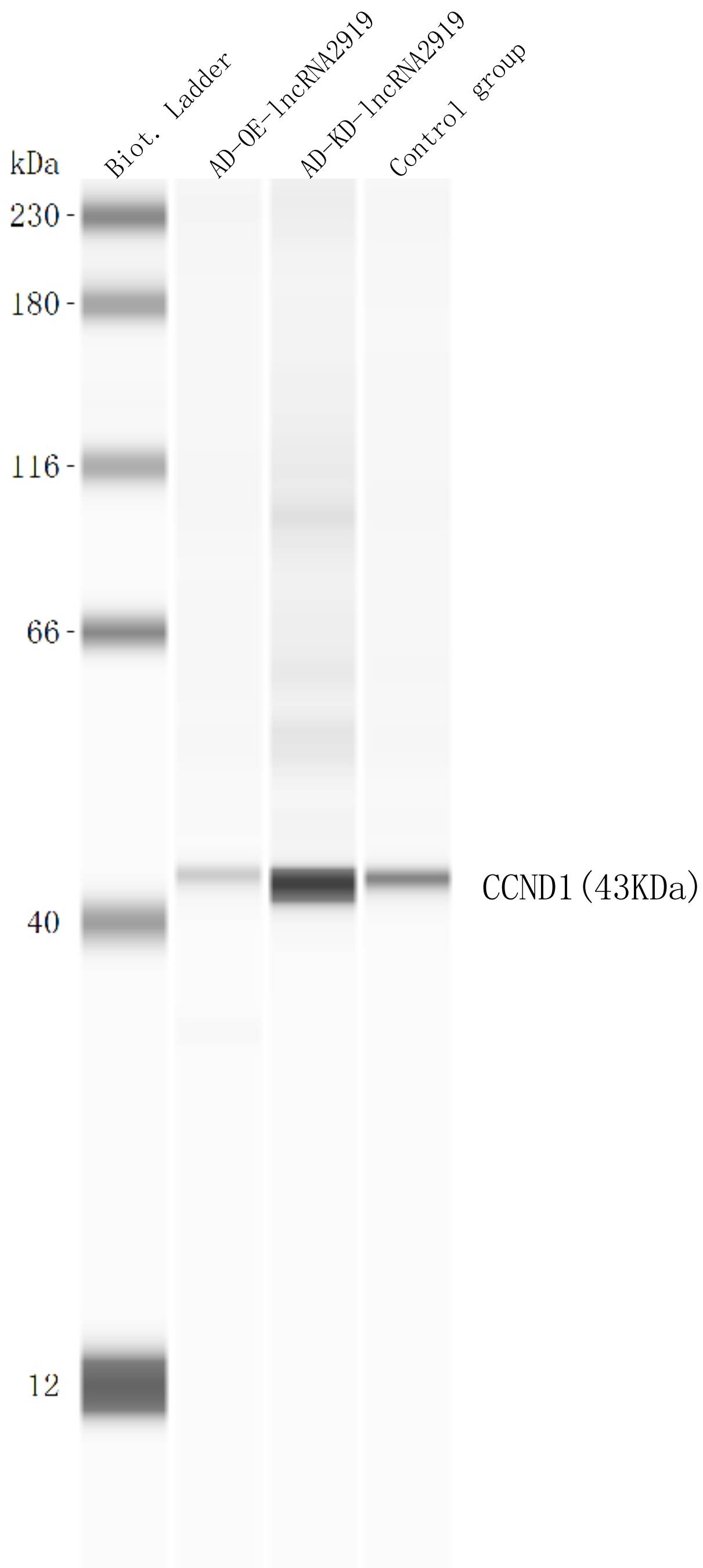

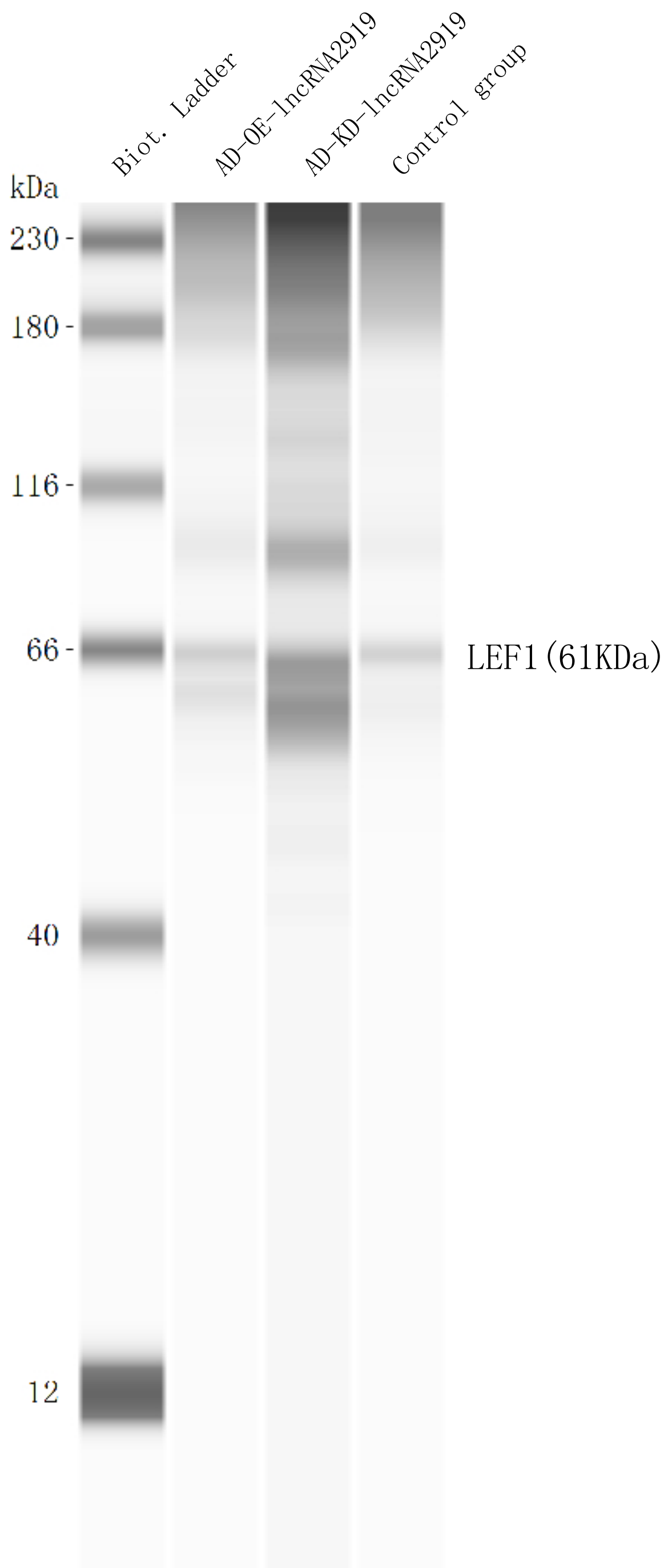

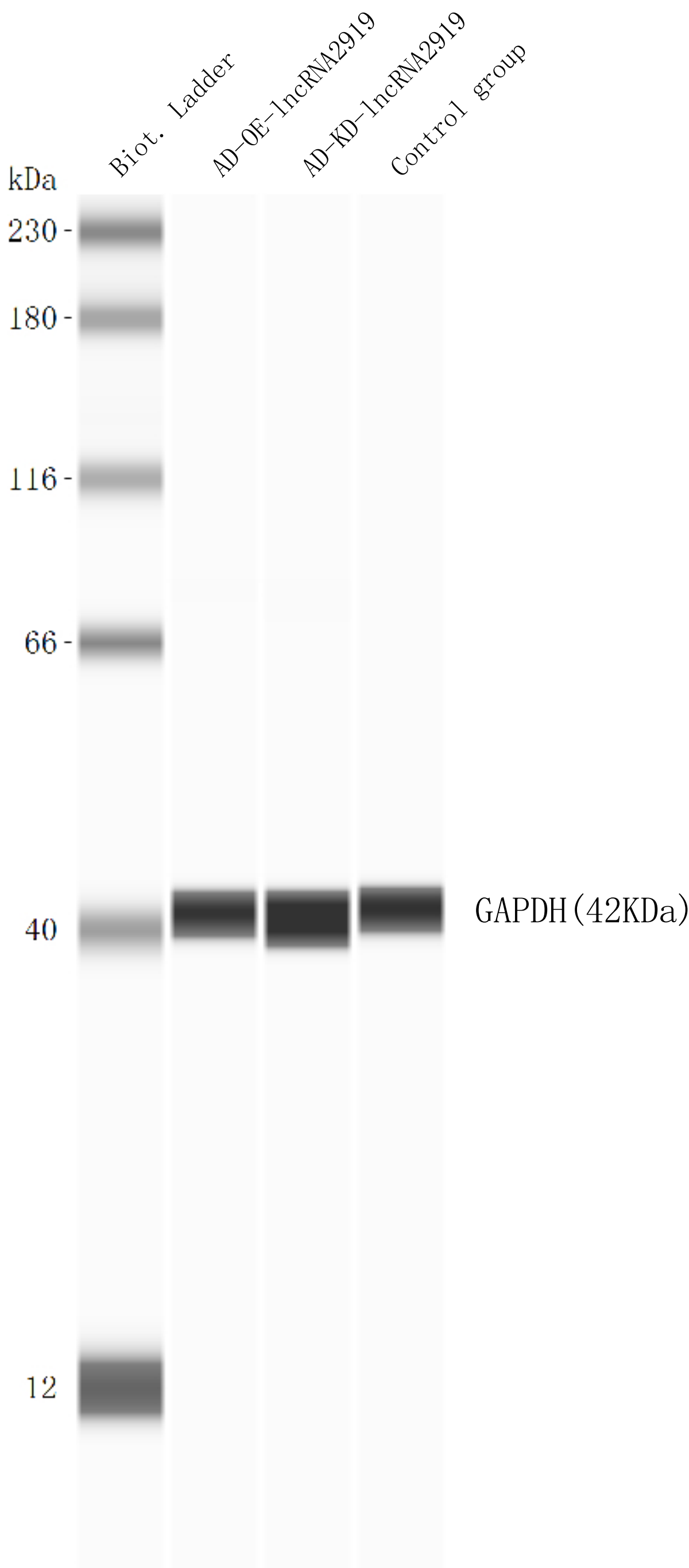

Supplement: Supplementary file 1 [file ijms-23-09481-s001.zip › Supplementary file S1.pdf]
